# Delineating the cognitive-neural substrates of writing: a large scale behavioral and voxel based morphometry study

**DOI:** 10.1038/s41598-019-55129-3

**Published:** 2019-12-11

**Authors:** Haobo Chen, Xiaoping Pan, Wai-Ling Bickerton, Johnny King Lau, Jin Zhou, Beinan Zhou, Lara Harris, Pia Rotshtein

**Affiliations:** 1 Department of Neurology, Guangzhou First People’s Hospital, School of Medicine, South China University of Technology, Guangzhou, 510000 P.R. China; 2 School of Psychology, University of Birmingham, Birmingham, B15 2TT UK; 3 School of Psychology and Clinical Language Sciences, University of Reading, Harry Pitt Building, Reading, RG6 7BE UK; 4 Faculty of linguistics, philology and phonetics, University of Oxford, Oxford, OX1 3UD UK; 5 Department of Psychological Medicine, King’s College London, London, WC2R 2LS UK

**Keywords:** Language, Motor control, Stroke

## Abstract

The current study investigated the cognitive and neural substrates that underpin writing ability. We explored similarities and differences in writing numbers and words and compared these to language and manual actions in a large group of sub-acute, stroke patients (n = 740). The behavioral data showed association and dissociation in the ability to write words and numbers. Comorbidities of writing deficits with both language and motor impairments were prevalent, with less than a handful showing deficits restricted to the writing tasks. A second analysis with a subset of patients (n = 267) explored the neural networks that mediate writing abilities. Lesion to right temporal contributed to writing words, while lesions to left postcentral contributed to writing numbers. Overlapping neural mechanisms included the bilateral prefrontal cortex, right inferior parietal, left middle occipital and the right cerebellum. With the former regions associated with error pattern typical to writing based on prior knowledge (the lexical route), while lesion to left MOG was associated with errors to the phonological (non-lexical) route. Using principle components extracted from the behavioral data, we showed that right prefrontal and right parietal contributed to the ability to use pen, while lesion to bilateral prefrontal, inferior temporal and cerebellum supported unique use of pen for writing. The behavioral and imaging data suggested that writing numbers and words primarily relied on overlapping cognitive and neural functions. Incidents of pure writing deficits, in the absence of motor or language deficits were rare. Nevertheless, the PCA and neural data suggested that writing abilities were associated with some unique neuro-cognitive functions, specifically dedicated to the use of pen and the ability to transform meaning to motor command.

## Introduction

Only Human beings have developed an ability to use visual marks (writing) as a mean of communicating ideas that overcome the boundaries of time and space. In contrast to spoken language, writing is a relatively recent addition to human behaviors. It is not an essential ability for human basic survival needs. Thus it is not commonly used or practiced by all human cultures^1^. Prominent cognitive models of writing suggest it is enabled through interaction between language processes and high-level manual motor control^2–6^. The current study aimed to provide a detailed cognitive and neural map of the writing ability, using neuropsychological data. We specifically consider the relation of writing to language and manual motor abilities. In addition, we explored whether different writing systems (words vs. numbers) are associated with overlapped or dissociated cognitive-neural mechanisms.

The working hypothesis of the current study is based on the neuronal recycling hypothesis^1^. According to this hypothesis, universal cortical specialized areas emerge to accommodate the acquisition of a new skill. The anatomical location of these regions is constrained by the functional architecture of the brain. This hypothesis has been demonstrated in visual associative cortices. Specifically, it is suggested the lateral banks of the middle occipital gyrus (the visual word form area, VWFA) ‘specializes’ in perceiving and reading visual symbols, independent of the writing systems^1^. A region anterior to the VWFA is hypothesized to specialize in reading numbers^7^. Most of the work, supporting the neuronal recycling hypothesis is based on functional MRI studies focusing on interpreting the visual input, aka reading. Here using neuropsychological data, we focused on the motor output, the production of meaningful visual signs and symbols, as required by writing.

Writing may broadly refer to all the activities involved in handwriting, printing, cursive writing, and typing, that are in responding to various kinds of input including visual, auditory, and one’s thoughts. In the current study, we explored writing processes in a narrow sense, focusing on the use of handwriting for single words, numbers or numerical symbols (e.g. ‘£’) in response to an oral cue, a dictation.

The act of writing involves multifaceted cognitive processes including but not limited, to linguistic related processes, assignment of meaning to visual-symbolic representations, eye-hand coordination, and high-level motor control. Goldstein^2^ proposed one of the first models for writing which is still accepted today. It suggests that linguistic and motor abilities contribute to handwriting^3,4^.

The formal neuropsychological syndrome associated with acquired writing deficits is agraphia^8,9^. There are different types of agraphia owing to the multi-facet nature of writing: central agraphia and peripheral agraphia. The central-peripheral subdivision parallels the theoretical notion that writing requires the interaction of language and sensory-motor-related processes.

Patients with peripheral agraphia typically show errors related to poor motor or visualization abilities^10,11^. This includes the correct ordering and execution of the effector-specific muscle movements required for expressing the letters^12,13^. Peripheral motor agraphia primarily manifests in poor and illegible letters and their configuration.

Central agraphia relates to the linguistic component of writing and is also referred to as aphasic agraphia^11^. Central agraphia referred to patients with deficits in the use/ordering of the letters in a word^10,14^. For phonological based writing systems (e.g. English), the central component of writing are hypothesized to be utilized by two parallel routes^15–17^: a phoneme-grapheme conversion route (non-lexical route, phonological agraphia) which can process words and non-words; and phonic-orthographic route (semantic lexical route, semantic agraphia) which utilizes prior knowledge of words^12,13^. In this context, spelling errors in exceptional words and a large number of phonological errors (writing the words as they sound) reflect impairment of the lexical route and intact non-lexical phonological route. While the error in exceptional and regular words that are not phonological reflect deficits to the semantic lexical route.

The classical clinical reference for agraphia made by Exner^18,19^, identified the left middle frontal gyrus (MFG) as the writing area, also known as Exner’s area. Few following case studies supported the role of left MFG in writing ability^20–22^. Resection of a nearby region, the left superior frontal gyrus (SFG) is reported to results in long term agraphia, in the absence of speech aphasia in 15 cases^23^. Roux and colleagues^24^ report that intra-operative stimulating of the left SFG in 6 out of 12 patients led to interference with writing with no effect on language and motor abilities^24^. Writing impairment was also induced in healthy following virtual lesions (i.e. transcranial magnetic stimulation) of the left SFG^25^. Lesions to the supplementary motor area within the superior frontal gyrus were also associated with agraphia^26^.

Beyond the left prefrontal cortex (PFC), other brain regions have also been implicated in writing. An autopsy study described agraphia patients with a lesion to left angular gyrus (AG) sparing the left PFC^27^. The specific involvement of the left angular gyrus in writing was supported by some later case reports^28–31^. Agraphia cases were reported following lesions to left supramarginal gyrus(SMG)^3^, left superior parietal^10^, insula^3,32^, the basal ganglia^33^ and the left posterior inferior temporal cortex (ITG)^34–36^. Presumably, lesion to the left ITG disrupted the function of the previously mentioned VWFA^37^, suggesting this area may also play a rule in writing and not just reading. It is worth noting that most of the agraphia cases above often show comorbidities of aphasia, reading disorder (alexia) or naming disorder (anomia); while motor deficits in these cases are not consistently reported.

An intra-operative study^6^ reported deficits in writing following electrical stimulation to specific foci of the left middle/superior temporal and supramarginal gyrus (SMG). The study highlighted the high comorbidity of language and motor deficits with writing. Out of the 24 patients tested, only 8 showed pure agraphia symptoms after stimulating a highly confined area, with no other language or motor impairments. Stimulating the above regions produced both lexical and non-lexical errors^24^.

Not surprisingly, many case reports highlight the important contribution of lesions to the dominant left hemisphere to agraphia. But, some studies suggest a contribution of the right (non-dominant) hemisphere to writing, in right-handed patients^38–40^. Cases with agraphia symptoms were reported following a lesion to the right midline occipital and parietal lobe^41–43^, right temporal occipital^39^ and right frontal^38^.

Dissociating the motor and the linguistic components of writing was the aim of two meta-analyses of neuroimaging data with healthy participant^12,44^. Both analyses show that writing tasks elicit activation in a large and distributed bilateral network. Across both meta-analyses, the linguistic components of writing (i.e. central) are shown to be associated with activation in the left lateral prefrontal cortex; left temporal-parietal cortex and right cerebellum. The motor component of writing is associated with activation in left superior and middle PFC, pre-central gyrus and left inferior parietal lobe. Responses of right cortical areas have also been reported in both meta-analyses.

Taken together, regions within the left PFC, the left parietal and left temporal cortices are repeatedly reported concerning writing tasks in both functional imaging and neuropsychological studies. However, additional regions within the right hemisphere, as well as the cerebellum, are suggested to play a role in writing. Though reports of their involvement are less consistent.

The variety of writing systems developed by different cultures raised the question of whether the different ways of mapping symbols to meaning are associated with different neuro-cognitive mechanisms. The two common writing systems are the phonological conversion of sounds (phonemes)-to-letters (graphemes, e.g. English) and a logographic conversion of ‘units of meaning’-to-symbols (e.g. Arabic numbers, currency symbols, Chinese characters). The neuronal recycling hypothesis^1^, mentioned above, posits that similar brain areas become specialized in reading (/writing) independent of the writing systems. On the other hand, the prevalent assumption dissociates numerical from literacy processing^45^, suggesting the writing numbers may rely on different structures than writing words.

Dissociations and overlaps between different writing systems have been explored extensively in the perception domain, i.e. reading. Fewer studies have examined this question in the motor domain, i.e. writing. Writing models are silent on this question. It can be postulated, that independent of the writing system, some shared processes are always required. These include mapping visual (or auditory) input or one’s thinking to graphic symbols, eye-hand coordination and higher-level motor control (motor output).

The inability to process numbers, including writing them correctly, is typically associated with acalculia syndrome^11^. Deficits in writing numbers are attributed to lesions to the inferior parietal regions^46,47^, frontoparietal connections^48^ and the left perisylvian area^49^. It is worth noting that as acalculia is considered a dissociated syndrome form agraphia, common neuropsychological models will predict minimal overlap between writing numbers and words.

Surprisingly there are only a few studies that attempt to directly compare reading/writing of numbers and reading/writing words. A developmental study with school children reports a high correlation in the ability to write and read Arabic numbers and single words^50^. This study suggests that reading/writing words and number uses overlapping cognitive abilities. In line with this, comorbidity of numerical and language deficits is often noted^51^.

The neuropsychological literature is biased toward cases who demonstrate functional dissociation. Thus single cases are reported of patients showing deficits in writing numbers and words, despite intact numerical abilities^52^. Single dissociation is reported for patients showing impairment in writing words/letters but not numbers^53–55^. For example, Han and collages^55^ reported a case of a patient with a large left hemisphere lesion. In comparison to healthy controls, the patients showed a writing-to-dictation impairment of words and numbers, though the impairment in numbers was more severe. An opposite selective dissociation for writing deficits has not been reported to our knowledge (inability to write number with preserve ability to write words). Though cases of acalculia, presumably including an inability to write numbers are reported in the absence of language deficits^56^.

## Current Study

The current study had two aims: (1) to re-test the linguistic-motor model for writing ability using a data-driven approach and function lesion mapping with formal statistical tests. (2) To explore the overlaps and dissociations of different writing systems: words and numbers. We further assessed evidence for the dual-route hypothesis concerned with the phonological writing systems.

We used function-lesion mapping to answer the above questions. The advantage of this approach over fMRI/PET studies is that neuropsychological studies provide evidence regarding causality (processes in area ‘a’ directly contribute to the measured skill) while fMRI studies only measure correlations.

Previous neuropsychological studies with agraphia patients or patients with number processing deficits reported results based on relatively small sample size, patients were often pre-selected based on symptoms (e.g. showing selective deficits), or lesion location (e.g. unilateral lesion to the left hemisphere). The mapping of the lesion to symptoms rarely applied formal statistical methods to assess the reliability of the results. Comorbidities were also rarely controlled for. Hence the ability to generalize the results of these studies beyond the single cases is limited.

To increase the generalizability of the results, we did not pre-select patients based on formal neuropsychological diagnosis or specific lesion location. We use an inclusive large sample of stroke survivors recruited at their sub-acute phase (3 months post-stroke). Furthermore, to increase the sensitivity of the analysis, we did not classify the patient based on formal diagnostic criteria, but instead used their performance on relevant tasks and explored patterns in the data with minimal a-priori assumptions.

The cognitive data was collected using the Birmingham Cognitive Screen (BCoS)^57^. The BCoS is a clinical tool designed as a bedside test to provide a comprehensive cognitive profile of the patient. It has a shallow but broad approach for assessment. It measures 5 cognitive domains within 1 hr. Writing is part of the assessments of the language and the number domains. To assess generic language abilities, we used picture naming, sentence construction, and reading. We also used reading numbers to assess the knowledge of autographic numerical representations. We used the imitation of meaningless gestures, the complex figure copy, and the multistep object use tasks to assess the ability to control high-level manual movements.

The rich and large-scale continues nature of the data enabled us to utilize a data-driven approach to test whether language and motor components could explain variability in writing abilities. In the first analysis (N = 740) we report correlations and comorbidities of the writing tasks (number and words) with the tasks assessing other language and high-level motor functions.

In a second analysis, using a sub-set of the data (N = 267), we map the function to the lesion, by combining the behavioral data with clinical neuroimaging data (CT) using Voxel-based Morphometry (VBM). We specifically explored neural correlates supporting writing abilities and how these related to generic language and motor capacities. We first assessed the lesion associated with the raw scores of writing performances (model 1). Then we assessed how correlations with writing changed when we controlled for linguistic and motor processes in the model (model 2). We used conjunction analysis and exclusive masking to tap into shared and dissociated words and number writing systems. We then explored whether lesions in these areas were associated with specific error types. In a third analysis (model 3), VBM was used with latent cognitive writing components identified by principal component analysis (PCA). Combining PCA with VBM analysis has been used successfully in the past with this database to answer questions relating to spatial attention, language and drawing^58–60^.

## Method

### Participants

The BUCS trial tested nine hundred and six patients using the BCoS battery, after being admitted to the hospitals across the West Midlands (United Kingdom), due to stroke^61^. Patients were recruited in acute and rehabilitation stroke units. The inclusion criteria were as follows: the patient should (1) be within 3 months of a confirmed stroke; (2) be judged by the clinical team to be able to concentrate for at least 30 minutes to enable the tests to be administered; (3) have sufficient understanding of English to follow the instructions, and (4) have given written consent to participate. The patients were assessed in a quiet room within the hospital or the University. At the time of testing the patients and the examiners were blind to the lesions induced by the stroke.

#### Analysis 1

For the first behavioral analysis we excluded patients who were not assessed on the two writing tasks due to fatigue or other reasons (N = 166). This has left us with a sample of 740 patients (see Table 1, for demographic details).

**Table 1:** Demographic data and correlations.

Variables			Corr. Word-Writing		Corr. Number-Writing	
	Analysis 1 (N ≤ 740)	Analysis 2 (N = 276)	Analysis 1 (N ≤ 740)	Analysis 2 (N = 276)	Analysis 1 (N ≤ 740)	Analysis 2 (N = 276)
Gender (M/F)	322/418	131/136				
Right/left handed	656/68	267/0				
	**mean, med (std)**	**mean, med (std)**	**r**	**r**	**r**	**r**
Age Years	69.24, 71 [13.94]	70.28, 73 [14.29]	−0.052	−0.137^£^	−0.170**	−0.188*
Education Years	11.46, 11 [2.74]	11.40, 11 [2.61]	0.172**	0.205^£^	0.073^£^	0.152^£^
Stroke-to-scan Days	6.74, 1 [14.48]	6.75, 2 [11.71]	−0.042	−0.011	−0.019	0.074
Stroke-to-BCoS Days	27.62, 19 [27.24]	23.32, 16 [20.92]	−0.106^£^	−0.067	−0.146**	−0.151^£^
Barthel Index	13.32, 14 [5.66]	13.83, 15 [5.33]	0.159**	0.194^£^	0.251**	0.240**
**Cognitive data**						
Orientation (max = 8)	7.46, 8 [1.41]	7.51 [1.34]	0.420**	0.400**	0.55**	0.519**
Picture naming (max = 14)	10.82, 12 [3.36]	10.23, 12 [3.91]	0.527**	0.592**	0.618**	0.640**
Sentence construction (max = 8)	6.94, 8 [1.88]	6.50, 8 [2.47]	0.418**	0.506**	0.579**	0.672**
Sentence reading (max = 42)	37.44, 41 [9.54]	34.98, 41 [12.72]	0.500**	0.520**	0.554**	0.596**
Number reading (max = 9)	7.57, 9 [2.56]	7.16, 9 [2.98]	0.549**	0.574**	0.696**	0.708**
Multi step object use (max = 12)	10.26, 12 [3.32]	9.91, 12 [3.58]	0.315**	0.331**	0.46**	0.486**
Meaningless gest imitation (max = 12)	9.42, 10 [2.81]	9.31, 10 [2.91]	0.4**	0.465**	0.527**	0.543**
Complex Figure Copy (max = 47)	34.48, 38 [11.52]	33.94, 38 [12.56]	0.362**	0.279**	0.492**	0.562**
**Writing tasks**						
Numb writing (max = 5)	3.75, 5 [1.70]	3.54, 5 [1.85]	0.633**	0.700**		
Word writing (max = 5)	3.02, 3 [1.75]	2.74, 3 [1.87]			0.633**	0.700**

^£^p < 0.05 uncorrected; **P < 0.05 corrected (uncorrected *p* < 0.05/26). Corr, Correlation with number or word writing; Analysis 1 – descriptive and correlation for the sample that contributed to behavioral analyses; Analysis 2 – descriptive and correlation for the sample that contributed to VBM analyses. Med, median; std, standard deviation; max, maximum score in the task; gest, gesture; Numb, number.

The number of patients contributing to each variable in Analysis 1, age, n = 740; Education, n = 722; stroke-to-scan, n = 477; stroke-to-BCoS n = 740; Barthel Index n = 733; Orientation, n = 733, Picture naming, n = 730; Sentence construction, n = 730; Sentence reading, n = 707; Number reading, n = 715; Multistep object use, n = 722; meaningless gesture imitation, n = 737; complex figure copy, n = 721; number and word writing, n = 740. In analysis 267 contributed to all analyses.

#### Analysis 2

**Figure 1:**
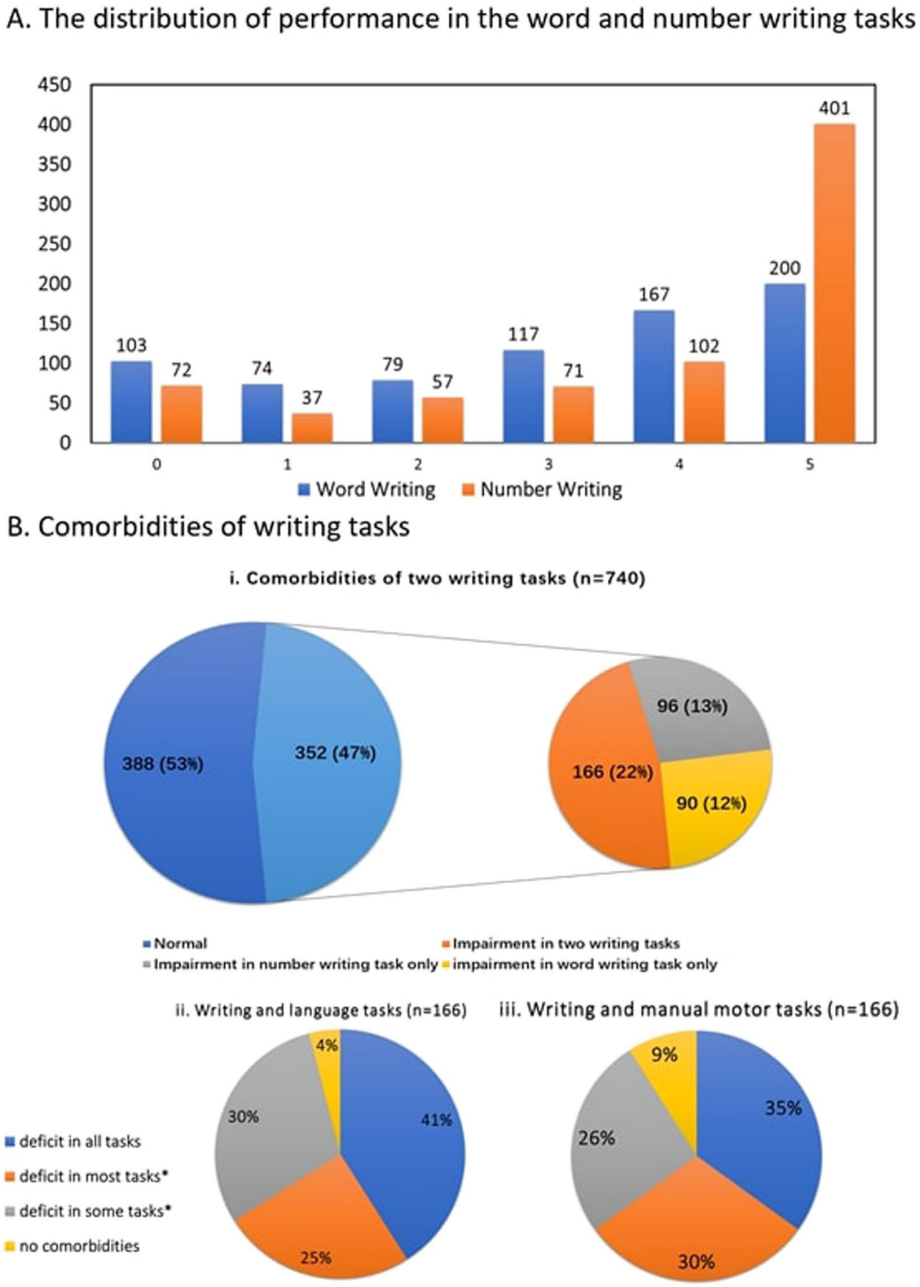
Behavioural results (N = 740). (**A**) The chart represents the distribution of performances for writing words (blue) and numbers (orange). (**B**) Pie charts representing the prevalence of writing impairments. The pie chart on the left, green represent patients who show no impairment in writing, blue represents patients who show impairment in at least one of the writing task. The pie chart on the right, break the blue group, to those how show impairment on both writing tasks (orange), or just on one of them (yellow, grey). (**C**) pie charts that break comorbidities in patients who show deficits in both writing tasks (the orange group) based on the prevalence of comorbidity with the language-based (left) and motor-based (right) tasks. *Deficit in most tasks (ii:3 language tasks; iii: 2 motor tasks); deficit in some tasks (ii: 1 or 2 language tasks; iii:1 motor task).

For the function-lesion mapping analysis, we first excluded patients who did not have a CT scan and those with poor quality CT scans or enlarged ventricles (N = 281). To reduce heterogeneity in our study, we also excluded patients with hemorrhagic lesions (N = 43) and left-handed patients (N = 76). Then we excluded 127 patients not assessed on the two writing tasks. Finally, as the evidence for ischemic stroke on a CT scan are unclear within the first 24 hours after stroke, we excluded those who had their scan taken on the same day of the stroke (n = 112). The analysis included a total of 267 ischemic stroke patients. (see Table 1 for demographic details of the two samples). Patients that were included in the second VBM analysis did not differ from those excluded in terms of age, gender, education year, Barthel index (all Ps > 0.05, see Supplementary Material Table 1 for details). We note that as this was an inclusive sample, the lesions cover the entire brain (see for overlap lesion maps based on the current sample: supplementary figure in Chechlacz’s paper^62^; Fig. 1 in Lau’s paper^60^), though were unevenly distributed as previously reported. For example, stroke affecting the middle cerebral artery (MCA) was much more prevalent than those affecting the anterior or posterior cerebral arteries (ACA, PCA), see details in Chechlacz’s paper^63^.

The BUCS study was approved by the UK National Research and Ethics Advisors’ Panel (NHS REC 08/H0301/6) and by the local Trust’s Research and Development departments in each hospital the patients were recruited from. According to guideline and regulation, all patients were informed on the purpose of the study and signed a consent form. The data is held anonymously and stored on secure servers (see for more details on the BUCS trial: http://www.bucs.bham.ac.uk/sites.php).

### Behavioral measures

#### Cognitive profile

The patients’ cognitive profile was assessed using the BCoS^57^. BCoS is a cognitive screen that measure performance across a broad range of cognitive abilities that can be classified into 5 domains using 23 tasks: (1) Attention and executive functions, (2) Language, (3) Memory, (4) Number Skills and (5) Action planning and control (Praxis). Here we used a subset of the tasks that were relevant to the current research question.

Cut-off scores, indicating significant impairment for each task are derived from 100 control participants without a history of a brain lesion. i.e. within the lower 5 percentile performances of the demographically aged-matched healthy controlled.

#### Word writing task

Patients are asked to write four familiar words and one non-word. There are two exceptional words – one concrete (‘scissors’) and one abstract (‘although’) – and two regular words, one concrete (‘mustard’) and one abstract (‘thinking’) and one non-word (‘troom’). The test assesses the ability to write with correct spelling based on phonological and lexical knowledge and the ability to control a pen. In the current study, we considered the writing performances across all words. The maximum score is 5, meaning that only words that were spelled with no errors and were recognizable were classed as correct. Participants who achieved an overall score of fewer than 3 points were classified as impaired in the task.

The writing sheets of 199 patients (out of 267) were available in the BUCS database. This enabled further analyses of errors in the writing word tasks. Error type analyses were conducted independently by two native English speakers, disagreements were resolved through discussions. For each patient, performances were coded for exceptional, non-exceptional and non-word. Phonological errors were defined as cases where the pronunciation was correct despite the wrong spelling. The writing quality of the letters was assessed, with a score of 2 indicating good writing quality, 1 recognizable with effort and 0 for those unrecognizable. Of the 199, 16 patients scored zero on the word writing task and their writing sheets were empty. These patients were excluded from the analysis.

Number Writing task, the patients are asked to write down two multiple digits numbers (e.g. 807) and three prices (£5.99). For the price writing, participants are required to present it clearly with a price symbol, i.e. ‘£’. Note that the reading task (see below) also included prices. It always precedes the writing task. This ensures patients are familiar with the pound symbol and the way the prices are expected to be written. In the case of patients fails to add the currency symbol, they are reminded once for each item that they are required to indicate that the number is a price.

The patient scored a point only for complete correct output, with a maximum score of 5. Participants who achieved an overall score of fewer than 3 points when aged 75 and older, or less than 5 when younger than 75, were classified as impaired in the task.

### Language correlated covariates

Picture Naming (PN) assesses the object naming ability^60^: The task consists of 14 line drawings of seven living things and seven non-living things.

In the Sentence Construction (SC) task, the patients are instructed to construct a sentence using two constraints: (1) the sentence must describe what a person is doing in the photograph shown; and (2) the sentence should contain two given words. The test measures whether the examinee has problems in semantic and syntactic processes. The maximum score was 8.

Sentence Reading task requires the reading of two sentences allowing the examiner to test the examinee’s ability to read different word classes (verbs, nouns, pronouns, adjectives, adverbs, and prepositions). There are both regular and exceptional words, as well as words with suffix and prefix in each sentence. The maximum score was 42.

Number Reading task requires the reading of three multi-digit numbers (e.g. 2,304), three prices (£109.50) and three digital times (e.g. 9:30). The maximum score was 9 points.

### Motor-related covariates

Like the writing task, these tasks were performed with the patient preferred hand, or the one that was least affected by the patients’ stroke.

In the Multi-Step Object Use Test (MOT) the patients are required to perform a sequence of actions with two objects (a battery and a torch, presented along with distractors) to complete a goal: light the torch. The task assesses patients’ ability to select the correct object, correctly interact with them and follow the sequence actions ending with the goal. The scoring discounted problems due to primary motor deficits. The maximum score was 12.

In the Meaningless Gesture Imitation (MI) test, the patients are required to mimic four meaningless manual gestures (including a sequence of two hand positions in relation to the head and two involve a single finger position). The maximum score was 12.

In Complex Figure Copy (CFC), patients are asked to copy a complex figure as accurately as possible. The scoring measures the organization of the figure and associated constructional apraxia as well as the presence of visual neglect^59^. The maximum score was 47.

### Other general covariates

Patient’s Orientation: there are 8 open-ended verbal questions to examine the patient’s ability to access personal information.

Barthel Index (BI): This used ten variables describing activities of daily living and mobility. A higher number indicates a greater likelihood of being able to live at home independently^64^.

In addition, we included the following measures as variables of no interest: age, gender, education, the use of the dominant hand and the time of the cognitive assessment relative to the stroke. See Table 1 for description.

### Analysis 1: behavioral data (N ≤ 740)

Analysis 1: To estimate the relation between the two Writing tasks and demographic data along with all the other covariates, Pearson’s correlation (two-tailed) analyses were performed. All together we computed 26 correlations, and the results were corrected for multiple comparisons using Bonferonni correction (p < 0.0019 (=0.05/26)). Table 1, present the correlation analysis. The data was not normally distributed, as evident by the difference between the mean and median (Table 1). When dealing with large datasets (N > 40) parametric statistical tests can still be used, even if it is not normality distributed^65,66^. Nevertheless, we also report in Supplementary Table 2 the correlation results using non-parametric statistics (Spearman correlation test). The pattern of results did not change.

Patients with missing data were excluded from the relevant analysis. For the comorbidities analyses (Fig. 1b), task impairments were defined using the cut off scores obtained from age and demographic match healthy control reported as part of the BCoS standardized data^57^.

### Analysis 2: neuroimaging assessment

The CT scans were acquired as part of the clinical routine when patients were admitted to the hospital. CT scans were acquired using Siemens Sensation 16, GE Medical System Light Speed 16 and Light Speed Plus with an in-plane resolution of 0.5 × 0.5 mm and a slice thickness between 4 and 5 mm.

### Pre-processing of brain images

We used SPM12 (Statistical Parametric Mapping) to preprocess the data in our study. The pre-processing steps were identical to the ones reported in previous studies using the BUCS data set^58–60^. In brief, DICOM data were first converted to NIFTI format. Consequently, we normalized the data by transforming images using an inhouse normalized CT template. Following this, we applied the SPM12 default unified segmentation algorithm (identical to the new segment in SPM8)^67^. The unified model is used to draw the deformable tissue probability maps (also called a-priori tissue class). The a-priori tissue maps indicate the probability of the voxel belonging to one of the six types of signals expected in a brain: GM (grey matter), WM (white matter), CFS (cerebrospinal fluid), bone, fat, and air. A 7th abnormal tissue type representing the lesion was also included^67^. We estimated that there would be a 10% probability of abnormal tissue in each grey/white matter voxel. The 10% was chosen based on the computed ratio of lesion volume to brain size from the same patients sample^58^. Finally, we applied a single Gaussian normal distribution to classify the intensity of the grey and white matter and two Gaussian distributions to classify the intensity of the abnormal tissue.

Quality checks: As described in previous studies using this database (e.g.^60,62^)we manually checked the quality of the segmentation and normalization of each image. This was done by uploading the segmented images (grey and white matter) of each patient, the a-priori segmented template, the normalized patient brain without the scalp and the canonical T1 provided by SPM. Then we checked the alignment of few key points: e.g. the most anterior and posterior parts of the corpus callosum, the edges of the cortex (most anterior/posterior, left/right lateral and ventral superior parts of the cortex), including the area surrounding the lesion. We excluded patients where the algorithm obviously failed (e.g. ventricles classified as white matter, the brain shape was deformed). Please refer to the Supplementary Figs. 2 and 3, for example of normalized CT images, wrapped using the estimated parameters of the unified segmentation algorithm. For examples of segmented CT images of patients from this database please refer to Chechlacz and Lau’s papers^60,68^.

Out of the 281 patients who were excluded from study 2 due to lack of brain images and poor quality image, 41 were excluded due to failure in segmentation. As mentioned in the text, this was primarily due to either bad quality CT or very large ventricles.

To accommodate the assumption of continuity for the random field theory, we smoothed the segmented GM by using a 12 mm^3^ FHWM Gaussian Kernel, to remove sharp edges. Using such a high filter is recommended for VBM studies when a simple model is used for wrapping^69^ (e.g. not DARTHEL). Finally, as the aim of the study was to identify stroke lesions that map to a function, it was expected effect size that span across a large number of voxels. According to the matched filter theory, the later assumption also entails the use of a fairly large filter for smoothing.

### Voxel-based morphometry (VBM)

To compute the correlation between the behavioral results of the word and number writing in relation to grey matter lesions, we used random-effects analyses within the general linear model framework^70^. Correction for multiple comparisons was done at the cluster level (p < 0.05, with uncorrected voxel threshold of p < 0.001).

We note that the analysis was done using the grey matter probability images, which means that sub-clinical atrophy may also contribute to the results. In the case of CT images, the grey matter tissue probability maps reflect the grey matter density. In other words, it was not done on lesion maps.

To reduce the potential impact that some demographic and clinical factors might have on cognitive performance and brain lesion, the following measures were included as covariates of no interest in all models: age, gender, years of education, interval between stroke and CT scanning, interval between stroke and cognitive testing, Barthel Index, ability to use the dominant hand and orientation.

#### Missing data

If data was missing in the covariates it was replaced by the group average. The amount of missing data for each task ranged from 0% to 5.6% with an average of 1.4%. While the number of missing data points was small, it was not equally distributed across impaired and intact writing groups. Patients who were impaired in the two writing tasks also had more missing data in the language task: 14 missing data points across the four tasks in the impaired group as opposed to 1 missing point in the intact group. Similarly, patients with writing deficits had 12 missing data points in the three motor tasks; while the intact group had only 4 data points missing. The approach we took to replace this missing data was a conservative approach, in which we replaced it by the average of the group. To ensure this did not lead to spurious results, we have re-run the PCA analyses (see below) excluding all patients with the missing data. The pattern of results has not changed; hence we kept the conservative approach for replacing the missing data. Though it is likely that the approach to replace missing data underestimated the prevalence of comorbidities.

In the VBM analyses, we estimated three models. Model 1 included the Writing (word + number) raw data with no additional language and motor covariates. Model 2 added the language and motor associated tasks as covariates. In both models: we report the shared lesions affecting the writing of numbers and words writing using conjunction with global null. The dissociated mechanism for Numbers’ and Words’ Writing was tested using exclusive masking. For example, we look for clusters that correlated with the ability to write Words (P_corr_ < 0.05), but did not correlate with the ability to write Numbers (voxel threshold: P_uncorr_ > 0.05). Function lesion mapping of error types: To explore the relations between lesions and specific cognitive function, we extracted the volumetric of the grey matter from 12 mm sphere clusters’ peak observed in model 2. These were correlated with the three error types: phonological errors, errors in exceptional and regular words and with the writing quality score (see above for details).

Model 3 was computed to gain further insights into the rule of different regions within the writing network. To this aim, we computed a PCA to identify underlying cognitive components for the two ‘Writing’ tasks. A KMO and Bartlett’s tests were performed across the data. The KMO value was 0.910 and its significance levels for the Bartlett’s test were all below 0.001 (1647.631 with 36 degrees of freedom). The KMO test results indicated that there was a correlation in the data selected and the distributions of data meet the assumptions of multivariate analysis.

The PCA was computed in SPSS, the data was scaled before the PCA was applied. The selection of tasks to be included in the PCA was driven by the Goldstein’s influential cognitive model for writing, which posits that writing represents an interaction between high-level motor and language processing. Therefore, the PCA included the two writing tasks (word and number), four language tasks (Picture Naming, Sentence Reading, Sentence Construction, number reading), and three motor-related cognitive tasks (Meaningless Imitation, Multi-object used and Complex Figure Copy). The PCA teased apart the differential and shared components of writing with the other cognitive tests. In brief, PCA aims to reveal latent variables by projecting the data onto a new space defined by the components. Each new component is a linear combination of the weighted original scores. Higher loading (weight) means a larger contribution of a specific task to this component. Model 3 included all the PCA components. The analysis of the PCA-VBM focused on components that were most clearly and meaningfully linked to latent variables associated with variability in Writing. The selection of components was based on their loading rather than overall explained variability. This was because neuropsychological studies are often based on case studies or pre-selected cases. These cases are at the tail of the distribution when considering the entire stroke population. Hence they are likely to explain a small amount of variability in the data when considering the entire stroke sample. This hinders the ability to generalize the current results beyond the current sample, as we discuss in study limitations.

**Figure 2:**
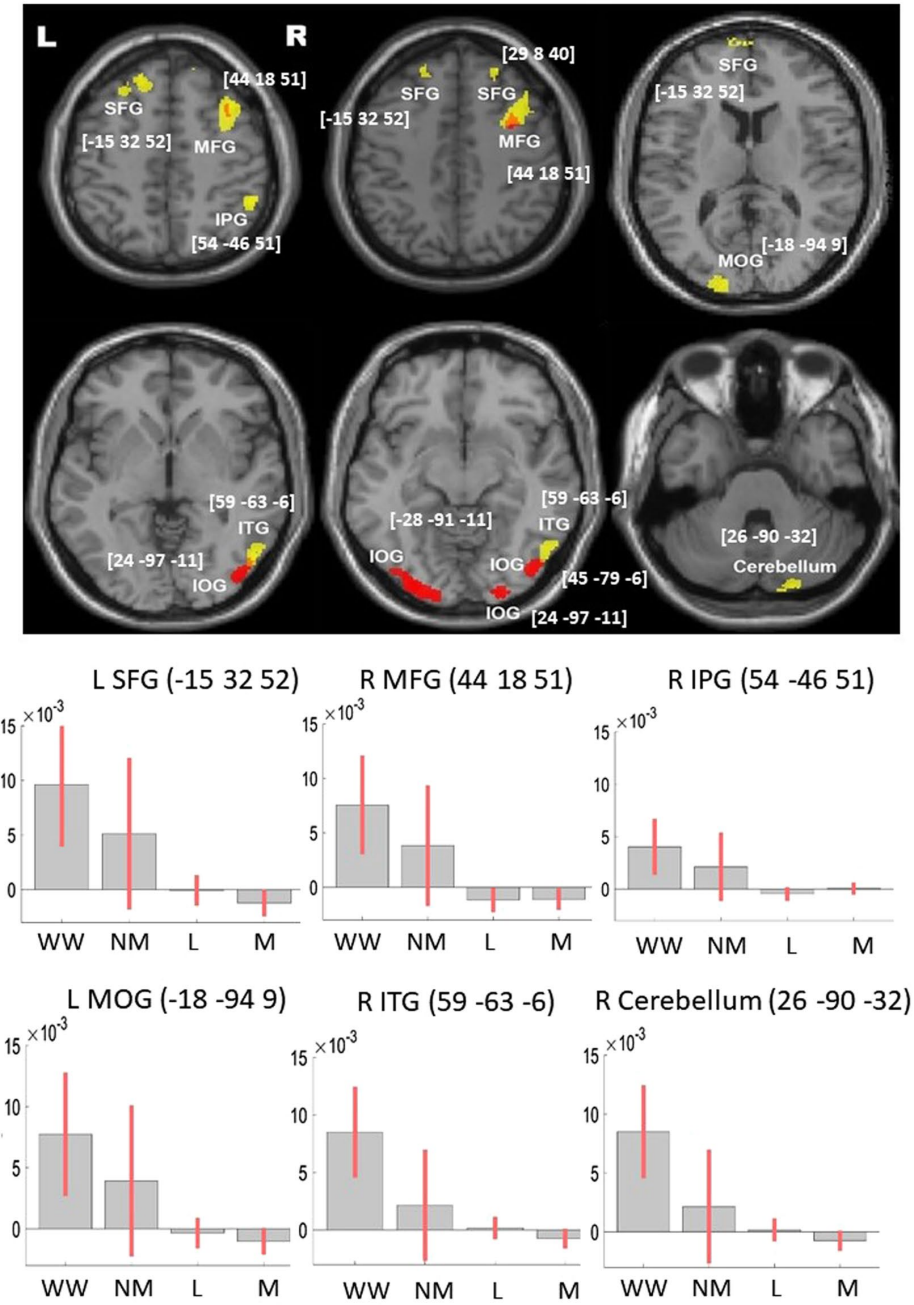
VBM results with raw scores (model 1 & 2) (N = 267). The VBM results overlaid on the canonical T1 images (SPM), showing lesions associated with deficits in both writing tasks (conjunction). Results of model 1 (model not included other language and motor tasks) are presented in red, and for model 2 (model included the 4 language and 3 motor tasks) in yellow. The charts represent effect size (beta) for each writing tasks and the average effect size for the language (L) and motor (M) tasks. The error bars are 90% confidence interval of the effect size. Abbreviations: L, left, R: right, SFG, superior frontal gyrus; MFG, middle frontal gyrus; IPG, inferior parietal gyrus; MOG, middle occipital gyrus; ITG, inferior temporal gyrus, IOG, inferior occipital gyrus; WW, word writing; NW, number writing; L, language, M, motor. See Supplementary Figs. 2 and 3 for case examples of lesion on the CT scans.

**Figure 3:**
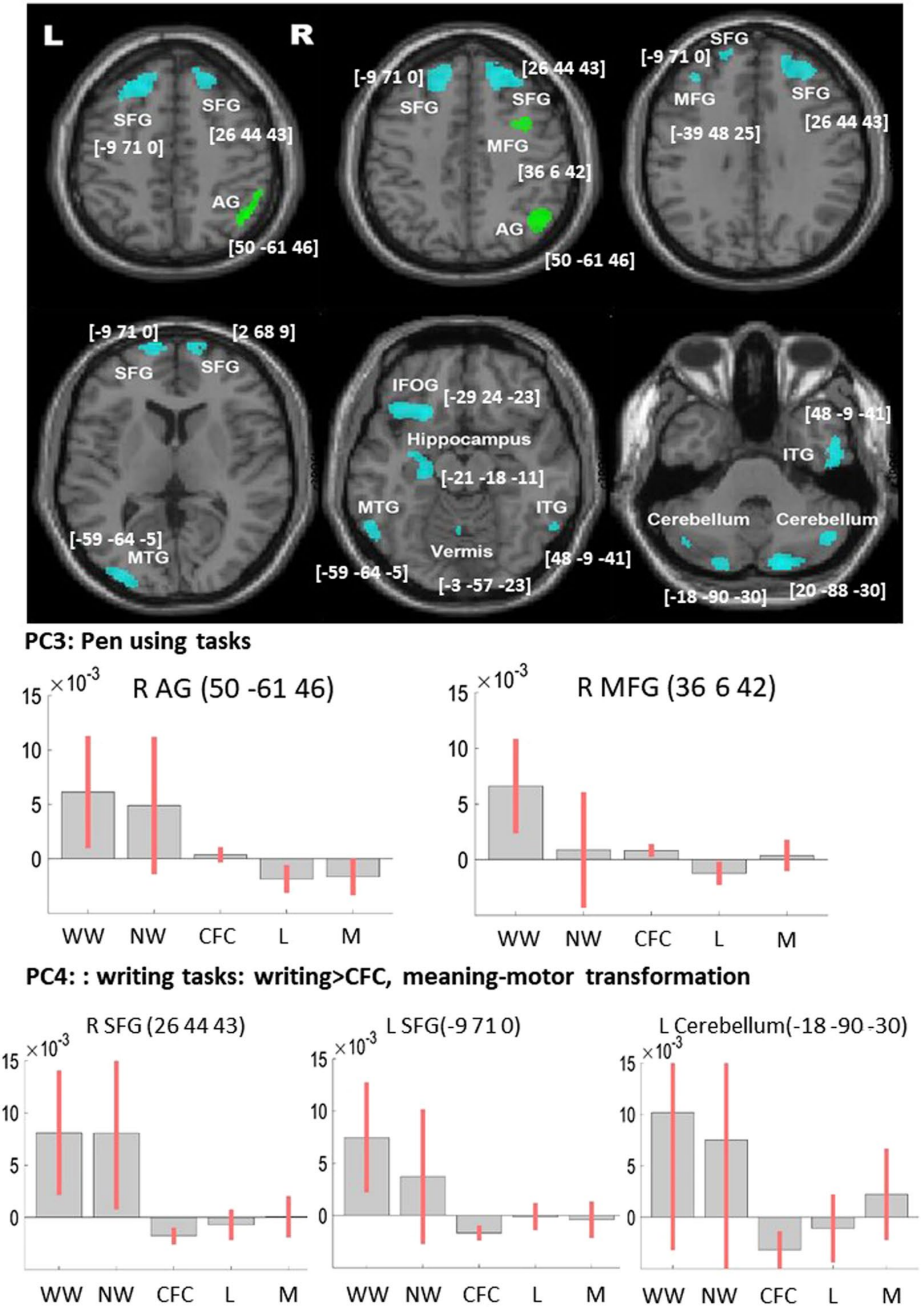
VBM results with PCA component (model 3, N = 267). The VBM results overlaid on the canonical T1 images (SPM), showing lesions associated with the PCA writing specific components. Lesion associated with using pen (PC 3) are presented in green, lesion associated with using pen to produce meaningful symbols (PC 4) are blue. The charts represent effect size (beta) for each writing tasks, the complex figure copy and the average effect size for the language (L) and motor (M) tasks. The error bars are 90% confidence interval of the effect size. Abbreviations: L, left, R: right, SFG, superior frontal gyrus; MFG, middle frontal gyrus; IOFG, inferior orbitalis frontal gyrus; AG, angular gyrus; MTG, middle temporal gyrus; ITG, inferior temporal gyrus, IOG, inferior occipital gyrus; WW, word writing; NW, number writing; CFC, complex figure copy; L, language tasks, M, motor tasks. See Supplementary Figs. 2 and 3 for case examples of lesion on the CT scans.

The charts in Figs. 2 and 3, represent the effect size (beta) for the covariates of interest, at the sphere of 3 mm around the peak. The covariates were scaled to ensure the betas are comparable.

## Result

### Analysis 1: behavioral results (N = 740)

Table 1 presents descriptive data for each of the measures and their correlation with the Word and Number Writing tasks. As can be seen, all the behavioral measures were positively skewed (median > mean), with more patients showing intact performances with a tail representing the severely impaired.

Word writing tasks- The patients analyzed in our study (=740) had an average score of 3.02 (SD: 1.75) in the Word Writing task (see Fig. 1A, for the distribution). 34.6% (256/740) of the patients were classified as impaired.

Number writing tasks- The average score was 3.75 (SD: 1.70) (see Fig. 1A, for the distribution). 35.5% (263/740) of the patients were classified as impaired.

Performance on word and number writing correlated (r = 0.63). Of the 740 patients, 352 (47.6%) showed deficits in at least one writing task. Of these 352 patients, 166 (22.4% of the 740) showed impairments in both word and number writing tasks, while 186 (25.1% of the 740) had dissociated abilities with 90 presenting deficits in word writing but intact number writing abilities and 96 presenting an opposite pattern (Fig. 1B.i). (see Supplementary Fig. 1 for the behavior results of the 267 patients recruited in the VBM analysis)

Correlation of the two Writing tasks with the clinical demographic data (Table 1) – both tasks showed a similar correlation pattern. As expected, the number of years in education positively correlated with the ability to write words. The Barthel Index had a weak positive correlation with both the writing tasks, indicating that patients with worse performance in activities of daily living were likely to struggle with writing. Age had a weak negative impact on the ability to write numbers, with older individuals performing worse than younger. As reported before with this sample, the number of days from stroke to test negatively correlated with the ability to write numbers. This is because the more severe patients were recruited from the rehabilitation center rather than from acute stroke units.

Correlation of the writing tasks with performances on the other cognitive domains- All the language-related tasks positively correlated with both the writing tasks (r ranged from 0.42 to 0.70). Similarly, all the motor-related tasks were positively correlated with performances on the writing tasks (r ranged from 0.32 to 0.53). See Table 1 for details.

The comorbidity data showed multiple dissociations of impairment patterns. None of the 740 patients showed impairment in the two writing tasks with intact abilities in all the other 7 tasks. Of the entire sample, thirteen patients (1.7%) were impaired only in writing words, and eight patients (1.08%) were impaired only in writing numbers. For the patients who were classified as impaired in both Word and Number Writing (n = 166), we counted the numbers showing impairments in the language or high-level motor domains. Of the 166 patients, only 7 (4%) patients show intact language ability, suggesting a specific writing impairment dissociated from language (Fig. B.ii), and only 13 (9%) patients had no other high-level manual deficit (Fig. 1B.iii). The most prevalent comorbidity was with complex figure copy (81.9%), though some patients (13.3%) showed deficits in writing but intact ability to copy a figure (4.8% of patients didn’t complete the copy task due to fatigue or other reasons). As writing requires the processing of graphemes we specifically examined whether patients with writing deficits were more likely to show deficits in reading tasks (reading words and Numbers) than in the speech tasks. Of those who had deficits in the two writing tasks (n = 166) only six showed deficits also in the reading tasks (sentence reading and number reading) but intact speech ability (picture naming and sentence construction), all these six patients were also impaired in the complex figure task.

The data above suggest that following stroke, the prevalence of writing deficits with no comorbidity of language and/or motor deficits is very low. The largest comorbidity by far was between tasks that require the use of pen, writing, and copying. As missing data was more prevalent in patients who were impaired in the writing tasks, the actual proportions of pure writing deficits may be even lower. Hence controlling for these comorbidities in the analyses is crucial.

Table 2 presents descriptive data of the error analysis for a sub-sample of patients (n = 183). Supplementary Figs. 2 and 3 provides case examples of impermanent lesion and error patterns observed in the data. There was large variability in performances. Patients struggled most with non-words and exceptional words. As the number of items in the writing words task was relatively small, we assessed the external validity of the writing measures using the error pattern.

**Table 2:** Error analysis writing words.

Error type	N	Score range	Mean/median/std	Number scoring zero
Writing quality	183	0–2	1.39/1/0.63	15 (8%)
Real words	168	0–4	2.09/2/1.34	30 (17.8%)
Non-word	168	0–1	0.35/0/0.48	109 (64%)
Regular words	168	0–2	1.4/2/0.79	32 (19%)
Exceptional words	168	0–2	0.68/0.5/0.76	84 (50%)
Phonological errors*	168	0–4	0.6/0/0.7	82 (48.8%)

*Correct pronunciation but wrong spelling, zero means no phonological errors.

The quality of writing was positively correlated with the three motor tasks that require fine manual control (Complex figure copy r = 0.431; Multistep object use, r = 0.388; meaningless gesture imitation, r = 0.245; all Ps < 0.001).

As expected, the number of errors made in words as opposed to non-word, correlated with the number of phonological error (r = 0.4, p < 0.001). The number of phonological errors negatively correlated with number writing ability (r = −243, p = 0.001). Similarly the number of errors in exceptional (relative to regular) words, positively correlated with number writing ability (r = −0.199, p = 0.011).

The final external validation test utilized relation pattern made by the dual-process model for writing^17^. This model predicts that the number of the error made in writing regular words can be predicted by the number of errors made on exceptional words (lexical route) and non-words (non-lexical route) (p(REG) = p(IRREG) + [1 − p(IRREG)] × p(NWD) (p = proportion, REG = regular word, IRREG = irregular word, NWD = Nonword))^17^. Based on this model, we computed for each patient a predicted number of errors for the regular words using their performances on exceptional words and non-word. The correlation of the predicted and the observed results was reliable r = 0.71, p < 0.001.

Taken together the error analyses demonstrated that despite the low number of items, the data present expected error pattern, supporting its reliability and external validity.

### Analysis 2: neuroimaging results (N = 267)

#### VBM based on raw scores of the writing tasks

The shared mechanisms for words and numbers writing were assessed using a global null conjunction. Dissociation between writing words vs. numbers was assessed using exclusive masks. Supplementary Figs. 2 and 3 provides case examples of impermanent lesions and error patterns observed in the data.

Model 1: First we examined the correlation of lesions with the raw scores of the Writing (word + number), without controlling for language and motor (Model 1, Table 3 and Fig. 2, red blobs). Lesions that predicted deficits in both the Writing tasks were in the right middle frontal and bilateral inferior occipital gyri. Model 1 showed no above threshold dissociations between writing numbers and words.

**Table 3:** VBM results – Analysis 2 (N = 267): function-lesion mapping of Writing.

Anatomy	Model 1		Model 2		Model 3: PC3 pen using	Model 3: PC4 writing vs. copying	
**Frontal lobe**							
L SFG	x y z cluster Zpeak		−15 32 52 580 4.31			−9 71 0 1906 4.94	
R SFG	x y z cluster Zpeak		2 68 9 150 4.07	26 44 43 219 3.88			2 68 9 150 4.07
L MFG	x y z cluster Zpeak					−39 48 25 187 3.33	
R MFG	x y z cluster Zpeak	29 8 40 250 3.63	44 18 51 952 4.02		36 6 42 450 3.65		
L IFG	x y z cluster Zpeak					−29 24 −23 844 4.66	
**Parietal lobe**							
R IPC/AG	x y z cluster Zpeak		54 −46 51 174 4.31		50 −61 46 628 4.88		
**Temporal lobe**							
L MTG	x y z cluster Zpeak					−59 −64 −5 2450 5.20	
L hippocampus	x y z cluster Zpeak					−21 −18 −11 698 4.16	
R ITG	x y z cluster Zpeak		59 −63 −6 445 4.17			48 −9 −41 462 5.12	59 −63 −6 445 4.17
**Occipital lobe**							
L MOG	x y z cluster Zpeak		−18 −94 9 459 3.91				
L IOG	x y z cluster Zpeak	−28 −91 −11 759 4.01					
R IOG	x y z cluster Zpeak	45 −79 −6 371 3.99	24 −97 −11 185 3.79				
**Cerebellum**							
L cerebellum	x y z cluster Zpeak					−44-72 −45 420 4.56	−18 −90 −30 424 4.18
R cerebellum	x y z cluster Zpeak		26 −90 −32 129 3.92			20 −88 −30 1146 5.34	45 −67 −45 604 4.35
Vermis	x y z cluster Zpeak					−3 −57 −23 553 4.21	

All reported clusters are family wise error corrected at the cluster level, with a voxel threshold of p < 0.001, uncorrected. Abbreviation: L: left; R: right; SFG, superior frontal gyrus/cortex; MFG, middle frontal gyrus/cortex; IFG, inferior frontal gyrus; IPC, inferior parietal cortex; AG, angular gyrus; ITG, inferior temporal gyrus; MTG, middle temporal gyrus; MOG, middle occipital gyrus; IOG, inferior occipital gyrus. Zpeak, the Z value of the cluster’s peak. Model 1 (light grey): cluster that positively correlated with number writing and word writing after controlling for age, education, orientation, days from stroke-to-scans, days from stroke-to-cognitive screen, gender. Model 2 (dark grey): Cluster that positively correlated with number writing and word writing after controlling for the covariates in model 1 and in addition for the 4 language and manual motor tasks. Model 3 (white background): Included the same covariates as in model 1, and the additional PCA scores as covariate. PC3, cluster showing positive correlation with the PCA component that dissociated the use of pen tasks from other fine manual motor tasks; PC4, Clusters showing positive correlation with the PCA component that dissociated the writing tasks from CFC (see Table 5 and text).

Model 2 controlled for the seven language and motor-related tasks (Table 3 and Fig. 2, yellow blobs). The previously observed clusters in bi-lateral inferior occipital were below threshold in model 2, suggesting variability in grey matter integrity in these regions could be account for by generic language and motor deficits.

Interestingly, by reducing the overall unexplained variability due to deficits in language and motor abilities, model 2 highlighted a correlation of the Writing tasks with lesions to bilateral superior frontal gyri, (SFG) right middle frontal gyrus, right inferior parietal and inferior temporal (ITG), left middle occipital gyrus (MOG) and right cerebellum.

Dissociation of writing words and numbers were observed, with a lesion to the right middle temporal (MNI: 58 −60 −2, Z_peak_ = 3.66, Voxels = 192, Cluster P_FWE_ = 0.023) uniquely associated with Writing words. This region was more posterior to the right ITG lesion observed for the conjunction analysis (Table 3). Conversely, lesion in the left postcentral gyri (MNI: −51 −19 45, Z_peak_ = 3.57, Voxels = 156, Cluster P_FWE_ = 0.061) was uniquely associated with Number Writing.

To gain a better understanding of the rule of each of these regions in writing abilities, especially writing words, we further examined the association of grey matter in the above cluster and the error types made by the patients (Table 4). The correlations were overall weak and hence should be interpreted with cautious. Lesions to the bilateral PFC and right inferior parietal were positively correlated with the ability to write exceptional words and negatively correlated with the number of phonological errors, suggesting a rule for these regions in the lexical route. While lesions to left MOG and right MTG positively correlated with the ability to write regular and exceptional words, suggesting a potential contribution to non-lexical processing.

**Table 4:** Error type & brain regions correlation.

Brain region (model 2)	Phonological errors	Regular words	Exceptional words
	*r, p*	*r, p*	*r, p*
L SFG	−0.20, 0.012		0.197, 0.012
L MOG		0.15, 0.049	0.15, 0.049
R SFG	−0.15, 0.055	0.17, 0.025	0.16, 0.040
R MFG		0.16, 0.036	0.15, 0.049
R IPC	−0.17,0.028		0.15, 0.049
R MTG	−0.176, 0.025		
R ITG	−0.167, 0.034	0.17, 0.034	
R Cerebellum			

Abbreviations: L, left, R, right, SFG, superior frontal gyrus; MFG, middle frontal gyrus; IPV, inferior parietal cortex; MTG, middle temporal cortex; ITG, inferior temporal gyrus. Results are not corrected for multiple comparison.

#### VBM based on PCA scores for writing

Model 3: To provide an alternative way of delineating the underlying cognitive components of Writing, we computed a PCA. The PCA included the nine writing, language, and manual control tasks (see methods) (Table 5). **PC1:** The first component was shared among all the 9 tests and explained 61.97% of the variability. We assumed that this component represented the overall cognitive ability, generic comprehension or stroke severity in general. **PC2:** The second component was mainly loaded on the motor-related tasks, differentiated them from the other linguistic tasks. This component explained 9.1% of the variability. It showed nearly no correlation with the two writing tasks. We assumed that this component represented the general motor vs. linguistic processes that contribute minimally to writing. **PC3:** The third component mainly loaded on the two writing tasks and complex figure copy, differentiated them from the other tasks. We speculated that it represented the use of pen and motor processes associated with writing and drawing. This component explained 7.73% of the variability in the data. It is also reliably correlated with the assessed quality of the writing (r = 0.281, p < 0.001). **PC4:** The fourth component dissociated the writing tasks from complex figure copy. This component explained 5.9% variability of the data. We assumed it primarily reflected processing associated with writing, but not copying. We interpreted it as cognitive processes associated with the translation of knowledge (e.g. meaningful symbols, objects) to motor programs, as this component was also loaded on the multi-step object used task. This component did not correlate with the quality of writing (r = 0.080, p = 0.286).

**Table 5:** PCA results – Analysis 2 (N = 267).

Tasks	PC1	PC2	PC3	PC4	PC5	PC6	PC7	PC8	PC9
WW	0.728	−0.087	**0.519**	**0.342**	−0.081	**0.200**	0.064	0.160	−0.029
NW	0.839	0.021	**0.274**	**0.185**	0.125	−**0.326**	0.017	−0.236	0.085
SR	0.832	−0.298	−0.166	−0.178	0.105	**0.186**	0.277	−0.038	0.187
NR	0.896	−0.224	−0.094	−0.129	0.031	−0.064	0.130	−0.042	−0.314
PN	0.859	−0.184	−0.110	0.017	−0.031	**0.239**	−0.363	−0.158	0.002
SC	0.847	−0.224	−0.242	0.045	0.064	−0.246	−0.144	0.286	0.067
CFC	0.671	0.377	**0.345**	−**0.517**	0.094	0.009	−0.081	0.074	0.015
MSO	0.619	0.601	−**0.312**	0.255	0.277	0.111	0.048	0.019	−0.032
MI	0.747	0.290	−0.166	−0.006	−0.566	−0.055	0.067	−0.022	0.035
Exp. Var.	61.97%	9.10%	7.73%	5.93%	4.95%	3.54%	2.94%	2.20%	1.65%

Abbreviation: WW: Word Writing; NW: Number Writing; SR: Sentence Reading; NR: Number Reading; PN: Picture Naming; SC: Sentence Construction; CFC: Complex Figure Copy MSO: Multi-step object use; MI: Meaningless Gesture Imitation.

The PCA did not reveal the dissociations of the writing tasks with respect to the language domain. For example, the data did not dissociate between tasks that rely on graphemes (reading and writing) and those that rely only on speech (picture naming, sentence constructions).

The last component of interest was **PC6**, it explained around 3.5% of the variability, differentiated Words from Numbers Writing. It specifically contrasted Word Writing and Picture Naming abilities against Number Writing and Sentence Construction. Thus uniquely dissociated writing words from writing numbers, but possibly tapping to an underlying cognitive mechanism that also dissociated these four tasks, e.g. single item trial (word writing and picture naming) as opposed to multiple items trial (number writing and sentence construction). As number writing involved the combination of symbols and multi digits number in a trial.

The results of model 3 are presented in Table 3 and Fig. 3. Based on our study objective, we only focus on the components that were loaded on the writing tasks. Low scores in the third component (PC3, representing poor pen use ability) were associated with lesions to the right middle frontal and the right angular gyri. We noted that the right middle frontal gyrus was also observed in model 1 and 2, above. This suggests that in the context of the writing, this region involves in the motor-related component of the tasks (pen-using).

The fourth component mainly loaded on word writing and differentiated it from complex figure copy. Low scores were associated with bilateral superior frontal, left middle frontal, bi-lateral temporal and bilateral cerebellum. These results were similar to the function-lesions observed in model 2. No above threshold lesions were found to be associated with component 6, which dissociated numbers from word writing.

## Discussion

The current study aimed to explore the cognitive neural substrates associated with the handwriting of words and numbers. To answer our two questions the data suggests that handwriting led to the development of bilateral networks of neural structures. This network supports the linguistic and the fine visual-motor control needed for using a pen to reproduce meaningful visual marks. This network was partly dissociated from the generic manual motor and language-based processing. The data supports the classical writing model, which views writing as the intersection between language and motor.

The data also provides some support for the two routes model for phonological writing. The data suggest that bilateral SFG and right IPC contributed to the lexical route, while left MOG potentially contributing the non-lexical route.

Finally, the second question concerned dissociations in different writing systems (phonological and no-phonological). In line with the neuronal recycling framework, we found that writing numbers and words are largely supported by overlapped neuro-cognitive systems. Though, dissociations do exist at the behavioral as well as the neural level. We first discuss the overlapping cognitive neural mechanisms involved in writing words and numbers, and then the evidence for dissociation.

Within one month post an ischemic stroke, slightly less than half of the patients in our study showed impairments in the Writing tasks (Fig. 1a) taken from the BCoS battery^57^. Considering that the analysis excluded patients who were unable to concentrate for at least 30 minutes or had severe limb paralysis, the incidence of writing impairment maybe even higher.

### Writing words and numbers

The ability of writing words and numbers correlated. Writing was also associated with other language and motor functions, as revealed by the correlation analyses and the prevalence of comorbid impairments (Table 1, Fig. 1b). Significant and positive relationships with the writing tasks were found with all the four language and three motor related tasks. More than half of the patients who showed deficits in writing had also deficits in other language tasks; while a similar proportion had difficulty in other higher level manual based motor tasks. None of the tested patients show deficits in both number and word writing tasks with no comorbidity of language and motor. And only a handful showed deficits restricted to the writing content (words/numbers).

The relatively high-level of symptom-associations may not be surprising given that writing required both motor and language cognitive processes^3,4^. The high prevalence of deficits in the writing tasks and the other cognitive tasks was also evident in the PCA, which suggested that most of the variability in patients’ performance could be explained by a single shared component (Table 5).

However, the PCA analysis (Table 5) highlighted two unique handwriting components, which explained together around 13.7% of the variability in patients’ performances. These components were dissociated from the other language and manual motor-based tasks. One component (PC3) differentiated the three pen-using tasks from all the other tasks. PC3 potentially represented the fine manual visual-motor control processes required for handwriting. A second component (PC4) differentiated the writing tasks from the complex figure copy. PC4 potentially reflecting the underlining transformations of graphemes (from meaning) and orthographic knowledge to manual actions/motor commands associated with writing, but not copying of meaningless figures.

The PCA components, comorbidity data and error analysis support the model positing that writing relies on high level motor control and language abilities. This accords with previous neuropsychological theories of agraphia, advocating that the syndrome should be divided into two sub-types: peripheral (sensory-motor) and core-linguistic.

Going beyond this model, the current study specifically highlighted the importance of hand-object interactions in writing abilities. The comorbidity analysis showed that more than 80% of patients who had deficits in the writing task were also impaired in the complex figure task. Similarly, in the PCA, this was represented by PC3 and PC4. PC3 dissociated tasks that require the use of pen while PC4 dissociated between hand using tasks that rely on prior knowledge (writing and multi-step object use) versus not requiring prior semantic (copying).

Surprisingly, The PCA did not reveal the dissociations of the writing task with respect to the language domain. For example, the data did not dissociate between tasks that rely on graphemes (reading and writing) and those that rely only on speech (picture naming sentence constructions). There was only weak evidence for a dissociation between logographic (numbers & symbol) and phonological (words) writing systems (PC6), as this was observed only for the writing and not for reading tasks. The comorbidity analysis and the error analysis did not show strong dissociations between the different language routes. Dissociations in the lesion pattern between processes related to different aspects of language were also weak and unclear (see below).

Deficits in the ability to use pen were manifested by poor performances in the Writing tasks as well as in the complex figure copy task (PC3). These were associated with lesions to the right middle frontal gyrus and right angular gyrus (Table 3, Figs. 2 and 3). We reported an association of lesion to these regions with deficits in the complex figure copy task previously when analyzing data from the same trial^59^.

A second network for writing (Table 3, Figs. 2 and 3) reflected the observed dissociation of the use of pen for writing (producing graphemes and adhering to prior orthographic rules) as opposed to copying “meaningless” figure (PC4). This network included the more commonly reported writing associated regions. The left superior and middle frontal gyrus, which overlap the classical Exner’s area^18–22,24,25,44^. The right cerebellum is consistently shown to be associated with writing tasks in a meta-analysis of fMRI studies^44^. And the left temporal cortices, which overlap the word-form area, typically reported for reading^1^. A further error analysis (Table 4) showed more details of the writing substrates. Lesions contributing to PC4 were associated with impairment to the lexical route, as will be expected from areas that support the shared processing of writing words and numbers. In contrast, the lesion affecting the left middle occipital gyrus led to increasing errors to both exceptional and regular words, suggesting its potential involvement in non-lexical processes. Though this region did not show an expected increase in the number of phonological errors

Taken together, the behavioral and brain data suggest a dissociation between the motor and the language component of writing. It stressed that writing specific deficits in proficient writers are more likely to emerge from impairment to hand-object interactions processes (execution and sequence retrieval). The support for the dual-route model for writing was weak, writing deficits associated with specific routes of language processing (e.g. lexical vs. phonological, or graphemes vs. phonemes) were less common.

The lack of support for a dissociation between phonological and lexical writing processes is inconsistency with previous studies. This may be caused by the following reasons: (1) previous studies confound familiarity with writing systems; for example, testing English and Chinese in native Chinese or native English speaker^45,71^. (2) Using Kanji and Katakana with Japanese participants is exceptional because here participants are familiar with both writing systems to the same degree; though the age of acquisition differs slightly, as children start with katakana and are gradually introduced to more kanji characters. While few cases were reported to show double dissociation; these dissociations were challenged when using a less biased recruitment procedures^72^. Hence it is possible with proficient writers dissociation in deficits are less clear.

The advantage of using numbers and symbols representing the logographic writing system, is numbers are as familiar as words, with similar age of acquisition. There have been few theoretical models that challenge the dual-route account, arguing that phonological and lexical processing contribute both to writing/reading interactively irrespective of the writing system^73^.

As mentioned in the introduction, the involvement of regions in the right hemisphere in writing has been reported before^38,39,41–43^. We provide now case examples of patients with the lesion to the right hemisphere that also showed impairment in the writing tasks (Supplementary Fig. 2). The involvement of regions of the right hemisphere (right IPG) specifically in the motor-related component of writing may link to reports associating the right hemisphere with constructional apraxia^74^. In support of this, a study by Ardila and Rosselli^38^ report writing errors of spatial and organization nature, following right hemisphere lesions. Further research will be needed to investigate in more detail the relations between constructional apraxia and writing.

Similarly, the involvement of the right inferior occipital cortex, right superior frontal and right inferior temporal in writing was not expected and their involvement in writing is only rarely reported in the literature^38,75^. Hence, we should exert caution when interpreting these findings. We can speculate a few reasons why we observed associations between right hemisphere lesions and writing. Regions in the right hemisphere are often recruited as a compensatory mechanism for reading. Studies have suggested the right hemisphere is more active with increased age^76^ and more active in poor readers^77^ and is involves in the acquisition of second language^78^. The right hemisphere has also been associated with wiring systems that are not phonological^79^. As the current sample was of relatively older adults, potentially included individuals with pre-stroke poor reading abilities and those that English was their second language; it could be that this is why lesions to their right hemispheres were also seen to impaired their writings. Future researches need to elucidate the exact contribution of these regions to writing abilities following stroke and the reproducibility of these observations.

### Writing words versus numbers

The comorbidity analysis suggested that the two different writing systems: for numbers and words had overlapping cognitive and neural architecture. About half of the patients who could not write words also could not write numbers. But double dissociations of writing words and numbers were also evident, where more than 50% of tested patients showed deficits in one writing system but preserved ability in the other (Fig. 1b). This double dissociation accords with the common model that numeracy and literacy are distinct processes^1,80^. In contrast, the correlation data and the data-driven approach analyses did not identify any component that dissociate numerosity (number tasks) from literacy (word tasks). Furthermore, we only observed a ‘weak’ component that dissociated numbers writing from words writing (Table 5: PC6). This component did not follow a numerical-literacy division, but grouped number writing with multiple object use task, and contrasted it against words writing and picture naming task.

The deficits analysis supports double dissociation, but the correlation, PCA and VBM analyses highlight overlapping processes. This contradiction may emerge because the deficits analysis is based on cut-offs and is ‘blind’ to the severity of the symptoms; while all others analyses use the continues scores and account for the severity of symptoms. This suggests that categorical divisions of data may amplify dissociations, which are potentially marginal.

The VBM analyses revealed reliable clusters representing shared words and number writing processes, see above. But there were also some dissociated structures, with lesion to the left post-central gyrus affected the ability to write numbers, while the lesion to the right inferior temporal interfered with the ability to right words. Previous fMRI study suggests that the left post-central gyrus is involved in processing numerical information^81^. A case of agraphia has been reported following a lesion to right temporal occipital cortices^39^. A study with poor reader individuals suggests that right temporal regions may be recruited as a compensatory mechanism to intact writing/reading abilities^82^. Though future research needs to assess the replicability of these observations and their specific rule in writing. Taken together, we suggest that the evidence for the dissociation between writing number and words are weak.

### Limitation and methodological consideration

We used data-driven approaches to delineate the motor and language components of agraphia, applying multiple analysis approaches. VBM-Model 2 controlled for the generic language and motor-related tasks. The analysis identified regions that specifically contribute to writing beyond generic language and motor abilities. Second, we used the words’ error types of data to investigate a specific rule of each region in writing. Model 3 used PCA data which enabled us to fine regions contributing to the fine motor or language component of writing separately.

By comparing English words and Arabic numbers, we introduced a new method for comparing logographic and phonological writing system while controlling for familiarity. By doing this we were able to show that there are minimal differences between the two writing systems as will be predicted by the neuronal recycling hypothesis.

PCA is a data-driven approach. Therefore, the interpretation of the components is speculative, it was based on the weighting of the tasks on the component. In order to validate the observed component structure, we reported the number of cases displaying the dissociation identified by the PCA analysis. However, we acknowledge that these interpretations should be taken with caution.

Correlation, VBM, and PCA assess linear parametric relations. As the data was not normally distributed, the results were likely to be driven by the tail of the distributions. We believe that this is appropriate given the nature of the data and the inclusivity in the way the patients’ population was sampled. Hence one would expect that the tail of the distribution representing the abnormal cases would primarily drive the results. Though it hinders that ability to generalized the component pattern beyond the observed data.

Any cognitive ability (e.g. writing) is likely to be impeded by deficits in core abilities, like sensory processing, working memory, executive function and attention (see for example Han’s paper^55^). The impact of these core abilities was potentially reflected by the first PC component, which explained most of the variability. The rule of the above core functions in writing is beyond the aim of the current study. We believe that by using the other cognitive tasks as controls (e.g. reading, copying), and the PCA analysis, we removed variability in writing due to deficits in these core abilities.

We assessed deficits in the current study using the BCoS, which adopt a shallow but broad approach. The shallow aspect means that a specific ability is assessed using a limited number of items. In the case of word writing, four words and one non-word are dictated; in the case of number writing 5 numbers are dictated. The detailed error analysis confirmed the reliability and external validity of the word writing task. The strength of BCoS is its broad approach, which means it provides a relative detail profile of cognition, which is not limited to one domain. The BUCS dataset provides a powerful research tool to assess the prevalence and comorbidity of deficits in a large and representative patient population. But the limitation is that it cannot replace formal clinical diagnosis of the known syndrome as it does not adhere to formal diagnostic criteria and has a relatively small number of trials per task. Hence our results reflect different components of writing deficits, but we cannot draw a direct conclusion on agraphia symptoms.

Finally, as with all clinical based data, the cognitive as well as the neural data are noisy as they are based on sub-optimal parameters (e.g. only five trials for assessing writing; density scans (CT) in different interval time, for assessing neural integrity). We believe that the large number of patients used here compensated for these relatively noisy measures. This is evident by the fact that most of our results replicated previously reported findings. However, we also observed a few regions that were unexpected. While most previously function-lesion mapping studies were based on pre-selected patients samples and single cases, it is difficult to assess the validity of the unexpected results. Future research would need to clarify the reliability of these findings.

## Conclusion

The current study identified two dissociable networks that have been specifically evolving to support writing: a visual-manual motor ability to use pen mediated by right angular and middle frontal gyri; and an ability to transform symbolic representations grapheme) to manual programs for used with pen. Lesions to the bilateral prefrontal cortex, left middle and inferior temporal and right cerebellum (among other regions) contributed specifically to writing. The latter regions are suggested to be primarily involved in lexical based writing. The study also supported a large overlap of number and word writing, though neuro-cognitive dissociations were also observed. The combination of detail description of behavioral performances alongside multiple analyses approaches for functional-lesion mapping enabled us to provide a compressive account of the cognitive-neural networks that support writing abilities.

## Supplementary information

Supplementary table and figure
