# Supplementary material for: Delineating the cognitive-neural substrates of writing: a large scale behavioral and voxel based morphometry study
Source: Sci Rep. 2019 Dec 11;9:18881. doi: 10.1038/s41598-019-55129-3 (PMC6906401; doi:10.1038/s41598-019-55129-3)

**Delineating the cognitive-neural substrates of writing: a large scale Behavioral and voxel based morphometry study**

Haobo Chen^a,b*^, Xiaoping Pan ^a*^, Wai-Ling Bickerton ^b^, Johnny King Lau ^b,c^, Jin Zhou ^a^, Beinan Zhou ^b,d^, Lara Harris^b,e^, and Pia Rotshtein ^b^

^a^Department of Neurology, Guangzhou First People's Hospital, School of Medicine, South China University of Technology, Guangzhou 510000, PR China

^b^School of Psychology, University of Birmingham, Birmingham, B15 2TT, UK

^c^ School of Psychology and Clinical Language Sciences, University of Reading, Harry Pitt Building, Reading, RG6 7BE, UK

^d^Faculty of linguistics, philology and phonetics, University of Oxford, Oxford OX1 3UD, UK

^e^ Department of Psychological Medicine, King’s College London, London WC2R 2LS, UK

Corresponding authors:

Haobo Chen, Department of Neurology, Guangzhou First People’s Hospital, 602 Renminbei Road, Guangzhou 510000, People’s Republic of China, Email: eychb@scut.edu.cn

Xiaoping Pan, Department of Neurology, Guangzhou First People’s Hospital, 602 Renminbei Road, Guangzhou 510000, People’s Republic of China, Email: qpanxp@163.com

Supplementary Table 1 demographic data comparison between study and excluded group.

|  | Age | Education year | Interval between CT and stroke | Interval between screen and stroke | Barthel index | Gender(Male) |
| --- | --- | --- | --- | --- | --- | --- |
| Study group | 70.28 ±14.29 | 11.41 ±2.65 | 6.76 ±12.30 | 24.02±21.18 | 14.49±12.12 | 49.1% |
| Excluded group | 69.88 ±13.57 | 11.35 ±2.75 | 6.18±15.35 | 34.27±70.48 | 12.65±5.82 | 43.4% |
| *p* | 0.091 | 0.185 | 0.206 | 0.011 | 0.482 | 0.122* |

Supplementary Table 2 spearman correlation tests between two writing tasks and other cognitive tasks

| ***Variables*** |  | | | ***Corr. Word-Writing*** | | | | ***Corr. Number-Writing*** | |
| --- | --- | --- | --- | --- | --- | --- | --- | --- | --- |
|  | | **Analysis 1**  (N≤740) | **Analysis 2**  (N=267) | | **Analysis 1**  (N≤740) | **Analysis 2**  (N=267) | **Analysis 1**  (N≤740) | | **Analysis 2**  (N=267) |
| Gender (M/F) | | 322/418 | 131/136 | |  |  |  | |  |
| Right/left handed | | 656/68 | 267/0 | |  |  |  | |  |
|  | | **mean, med (std)** | **mean, med**  **(std)** | | **r**  **Spearman** | **r**  **Spearman** | **r**  **Spearman** | | **r**  **Spearman** |
| Age Years | | 69.24, 71 [13.94] | 70.28, 73 [14.29] | | -0.078 | -0.135^£^ | -0.210** | | -0.188* |
| Education Years | | 11.46, 11 [2.74] | 11.40, 11 [2.61] | | 0.160** | 0.157^£^ | 0.175** | | 0.152^£^ |
| Stroke-to-  scan Days | | 6.74, 1 [14.48] | 6.75, 2 [11.71] | | -0.001 | 0.041 | -0.195** | | 0.074 |
| Stroke-to-  BCoS Days | | 27.62, 19  [27.24] | 23.32, 16  [20.92] | | -0.123^£^ | -0.081 | 0.146** | | -0.151^£^ |
| Barthel Index | | 13.32, 14  [5.66] | 13.83, 15 [5.33] | | 0.155** | 0.223** | 0.260** | | 0.240** |
| **Cognitive data** | | | | | | | | | |
| Orientation (max=8) | | 7.46, 8 [1.41] | 7.51, [1.34] | | 0.404** | 0.369** | 0.485** | | 0.476** |
| Picture naming (max=14) | | 10.82, 12 [3.36] | 10.23, 12 [3.91] | | 0.466** | 0.597** | 0.528** | | 0.613** |
| Sentence construction (max=8) | | 6.94, 8 [1.88] | 6.50, 8 [2.47] | | 0.336** | 0.441** | 0.502** | | 0.627** |
| Sentence reading  (max=42) | | 37.44, 41 [9.54] | 34.98, 41 [12.72] | | 0.570** | 0.608** | 0.514** | | 0.606** |
| Number reading  (max=9) | | 7.57, 9 [2.56] | 7.16, 9 [2.98] | | 0.508** | 0.592** | 0.636** | | 0.673** |
| Multi step object use (max=12) | | 10.26, 12 [3.32] | 9.91, 12 [3.58] | | 0.281** | 0.316** | 0.47** | | 0.480** |
| Meaningless gesture imitation (max=12) | | 9.42, 10 [2.81] | 9.31, 10 [2.91] | | 0.355** | 0.420** | 0.482** | | 0.510** |
| Complex Figure Copy (max=47) | | 34.48, 38 [11.52] | 33.94, 38 [12.56] | | 0.346** | 0.486** | 0.486** | | 0.559** |
| **Writing tasks** | | | | | | | | | |
| Numb writing  (max=5) | | 3.75, 5 [1.70] | 3.54, 5 [1.85] | | 0.568** | 0.652** |  | |  |
| Word writing (max=5) | | 3.02, 3 [1.75] | 2.74, 3 [1.87] | |  |  | 0.568** | | 0.652** |

Supplementary figure 1 Behavioral results of the 267 ischemic stroke patients


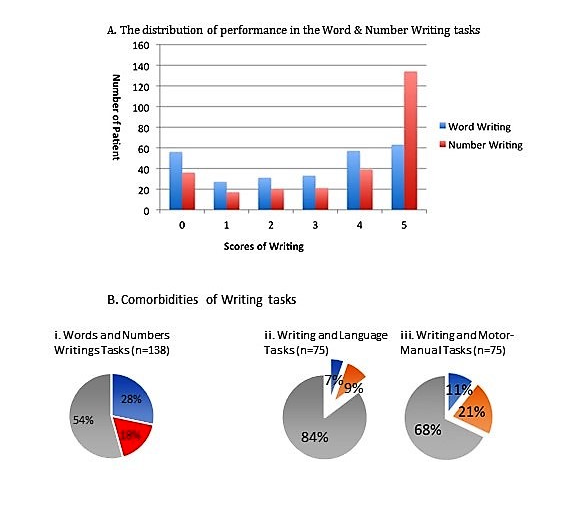


Supplementary figure 2 Case examples of impermanent lesion on the CT scans


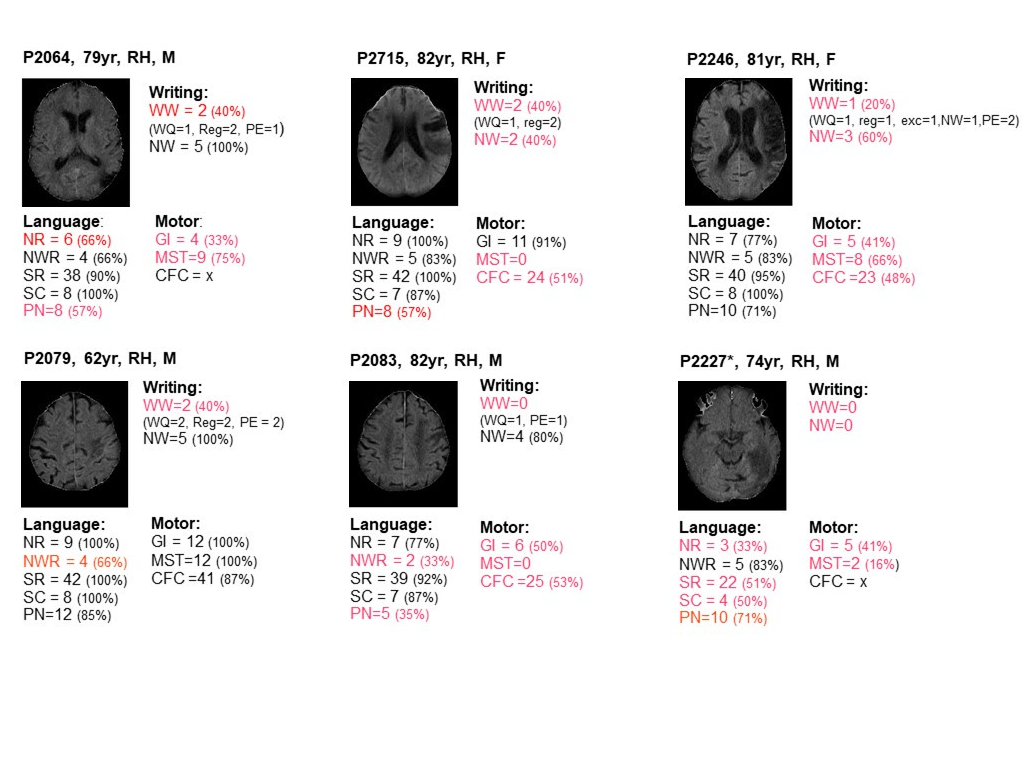


Supplementary Figure 3 Case examples of impermanent lesion on the CT scans
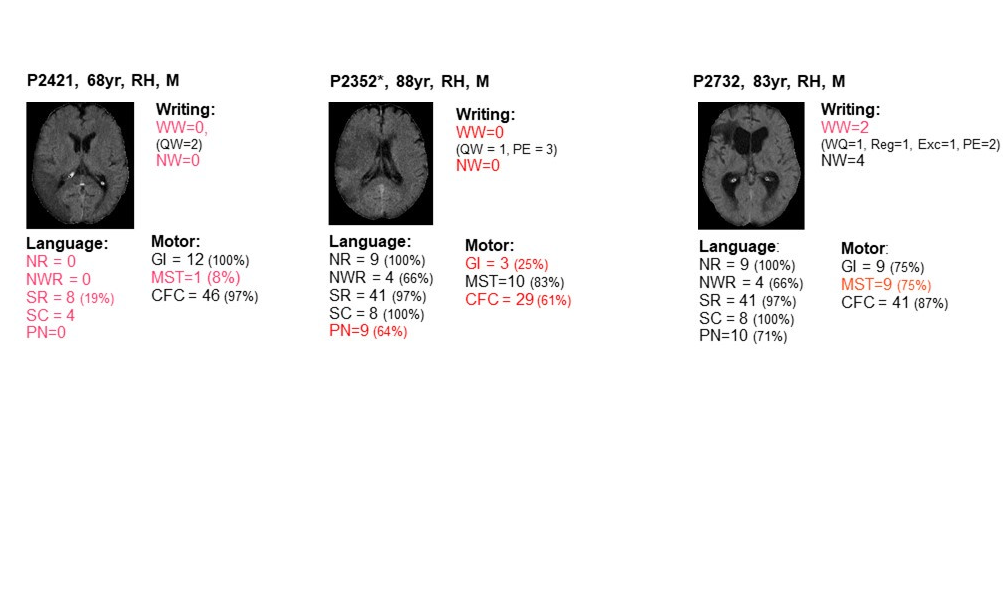

Supplement: Supplementary file 1 — Supplementary table and figure [file 41598_2019_55129_MOESM1_ESM.docx]
